# Multi-population genomic prediction using a multi-task Bayesian learning model

**DOI:** 10.1186/1471-2156-15-53

**Published:** 2014-05-03

**Authors:** Liuhong Chen, Changxi Li, Stephen Miller, Flavio Schenkel

**Affiliations:** 1 Department of Agricultural, Food and Nutritional Science, University of Alberta, Edmonton, AB T6G 2P5, Canada; 2 Department of Animal and Poultry Science, University of Guelph, Guelph, ON N1G 2W1, Canada; 3 Agriculture and Agri-Food Canada, Lacombe Research Centre, 6000 C&E Trail, Lacombe, AB T4L 1W1, Canada

**Keywords:** Multi-task learning, Bayesian model, Multi-population, Genomic prediction, Stochastic search variable selection

## Abstract

**Background:**

Genomic prediction in multiple populations can be viewed as a multi-task learning problem where tasks are to derive prediction equations for each population and multi-task learning property can be improved by sharing information across populations. The goal of this study was to develop a multi-task Bayesian learning model for multi-population genomic prediction with a strategy to effectively share information across populations. Simulation studies and real data from Holstein and Ayrshire dairy breeds with phenotypes on five milk production traits were used to evaluate the proposed multi-task Bayesian learning model and compare with a single-task model and a simple data pooling method.

**Results:**

A multi-task Bayesian learning model was proposed for multi-population genomic prediction. Information was shared across populations through a common set of latent indicator variables while SNP effects were allowed to vary in different populations. Both simulation studies and real data analysis showed the effectiveness of the multi-task model in improving genomic prediction accuracy for the smaller Ayshire breed. Simulation studies suggested that the multi-task model was most effective when the number of QTL was small (n = 20), with an increase of accuracy by up to 0.09 when QTL effects were lowly correlated between two populations (ρ = 0.2), and up to 0.16 when QTL effects were highly correlated (ρ = 0.8). When QTL genotypes were included for training and validation, the improvements were 0.16 and 0.22, respectively, for scenarios of the low and high correlation of QTL effects between two populations. When the number of QTL was large (n = 200), improvement was small with a maximum of 0.02 when QTL genotypes were not included for genomic prediction. Reduction in accuracy was observed for the simple pooling method when the number of QTL was small and correlation of QTL effects between the two populations was low. For the real data, the multi-task model achieved an increase of accuracy between 0 and 0.07 in the Ayrshire validation set when 28,206 SNPs were used, while the simple data pooling method resulted in a reduction of accuracy for all traits except for protein percentage. When 246,668 SNPs were used, the accuracy achieved from the multi-task model increased by 0 to 0.03, while using the pooling method resulted in a reduction of accuracy by 0.01 to 0.09. In the Holstein population, the three methods had similar performance.

**Conclusions:**

Results in this study suggest that the proposed multi-task Bayesian learning model for multi-population genomic prediction is effective and has the potential to improve the accuracy of genomic prediction.

## Background

Genomic prediction has become a new tool for selection of candidates based on genomic estimated breeding values (GEBV) through the use of dense markers covering the whole genome [1]. To predict GEBV, a training data set with genotypes and phenotypes is used to derive the prediction equations, where all marker effects are estimated simultaneously. GEBV for selection candidates that have genotypes are then predicted by summing up all the marker effects. The accuracy of GEBV is affected by several factors [2,3], of which the number of individuals in the training data set and the marker density are of crucial importance [2,3].

In Holstein dairy cattle, genomic prediction has been successfully applied using the Illumina BovineSNP50 single nucleotide polymorphism (SNP) panel [4,5]. For smaller populations such as Ayrshire in dairy cattle, acquisition of a large number of animals to be included in the training data set for genomic prediction still remains a challenge. One strategy is to combine data of the small populations with data from other populations to increase the size of the training set. However, simply pooling data from different populations may result in unfavorable accuracies if the marker density is low or the populations have diverged for a long time [6-8]. Increasing the marker density is one of the possible solutions because the linkage disequilibrium (LD) phase persistence between markers and quantitative trait loci (QTL) among different populations would likely to improve. However, a recent study in Jersey and Holstein dairy cattle reported a very limited advantage by using a very high density SNP panel [9]. Few studies have been dedicated to research new methods or strategies other than simply pooling data together for genomic prediction. Brondum et al. [10] proposed an approach called BayesRS for multi-population genomic prediction, where a location specific genetic variance derived in one population were used as priors for another population. They found that for some traits, BayesRS might be advantageous compared to the approach of simply pooling training data sets for distantly-related populations; but for closely related populations the method did not perform better than simply pooling data together.

Multi-task learning, the term first coined by R Caruana [11], aims to improve learning performance by simultaneously learning from related tasks. In text and speech recognition, image reconstruction and many other areas where data are collected from multiple sources, multi-task learning has been successfully applied [12-14]. Recently, the multi-task learning has attracted a growing interest in biological science for sequence and gene expression analyses [15-17] as well as genome-wide association studies (GWAS) [18]. To our knowledge, the application of multi-task learning in genomic selection has not been reported so far.

Bayesian learning models using stochastic search variable selection (SSVS) has been widely used and proven effective for genomic prediction in a single population [1,19-21]. Normally, SSVS uses some types of spike and slab distributions as priors for SNP effects. A latent indicator variable (0 or 1) is associated with each SNP, with 0 indicating that the SNP is irrelevant to the trait and is excluded from the model, and 1 indicating that the SNP is associated to the trait phenotype and is included in the model. In this study, it was assumed that multiple populations share the same set of latent indicator variables which can be learned jointly. The goal was to develop a multi-task Bayesian learning model for multi-population genomic prediction and to evaluate its performance on both simulated and real data.

## Methods

In this section, single-task Bayesian learning model, the simple data pooling method, and the multi-task Bayesian learning model were introduced. A Gibbs sampling algorithm was designed to implement the multi-task Bayesian learning model. Monte Carlo simulation studies were conducted to evaluate the performance of the proposed multi-task model. Real data on five milk production traits from Holstein and Ayrshire dairy breeds were also used to test the effectiveness of the multi-task model.

### Single-task bayesian learning model

In a single reference population of *n* animals with genotypes on *m* SNP markers, the statistical model can be written as:

$$y_{i}=\mu +\sum_{j=1}^{m}x_{\mathrm{ij}}a_{j}+e_{i},$$

Where *y*_*i*_ is the phenotypic value for the *i*^*th*^ animal (*i* = 1,…,*n*), μ is the intercept, *x*_*ij*_ is the genotype for the *j*^*th*^ SNP locus (*j* = 1,…,*m*) of the *i*^*th*^ animal, which is coded 0, 1 or 2, depending on the number of copies from a specified allele, *a*_*j*_ is the regression coefficient for the *j*^*th*^ SNP (allele substitution effect), and *e*_*i*_ is the random residual error.

A flat prior distribution is assigned to μ. *a*_*j*_ is assumed a mixture of a normal distribution $N\left(0,\sigma_{a}^{2}\right)$ and a point mass density at zero (denoted by a Dirac delta function *δ*_0_(*a*_*j*_) hereinafter). The weights for the two distributions are (1-w) and *w*, respectively, so that $\left(a_{j}|w,\sigma_{a}^{2}\right)\sim \left(1-w\right)N\left(0,\sigma_{a}^{2}\right)+w\delta_{0}\left(a_{j}\right).\;w$ follows a uniform prior distribution. A latent indicator variable γ_*i*_ is introduced for each SNP, so that when γ_*i*_=*1* , $a_{j}\sim N\left(0,\sigma_{a}^{2}\right)$ , and when γ_*i*_*=0* , *a*_*j*_=0. Prior distribution for each *γ*_*i*_ is assumed i.i.d. and follows Bernoulli distribution with probability (1-*w*). So the joint prior density for γ is $f\left(\gamma |w\right)=\prod_{j}w^{\left(1-\gamma_{j}\right)}{\left(1-w\right)}^{\gamma_{j}}$. Residual errors are assumed from a multivariate normal distribution $N\left(0,I\sigma_{e}^{2}\right)$ . The prior distribution for $\sigma_{a}^{2}\;\left(\sigma_{e}^{2}\right)$ is a scaled inverse Chi-square distribution with degree of freedom *v*_*a*_*(v*_*e*_*)* and scale factor $s_{a}^{2}\left(s_{e}^{2}\right)$.

### Simple data pooling method

Suppose animals are from a number of *c* different populations. In a simple data pooling method, animals from multiple populations are pooled together to form a single training data set. It is assumed that the population origin for each individual is known prior to the analysis. Population origin is included as a fixed effect. The effect of each SNP is assumed to be the same across populations.

### Multi-task Bayesian learning model

For *c* populations with *n*_*k*_ animals in the k-th population*,* the statistical model can be written as:

$$y_{\mathrm{ik}}=\mu_{k}+\sum_{j=1}^{m}x_{\mathrm{ijk}}a_{\mathrm{jk}}+e_{\mathrm{ik}}\;\left(i=1,\cdots ,n_{{}_{k}}\;\mathrm{and}\;k=1,\cdots ,c\right),$$

In matrix notation, this can be written as:

$$y_{k}=\mu_{k}1+X_{k}a_{k}+e_{k},$$

where *y*_*ik*_ is the phenotypic value for the *i*^*th*^ animal in the *k*^*th*^ population; *μ*_*k*_ is the general mean of population *k*; *x*_*ijk*_ is the genotype for the *j*^*th*^ SNP locus of the *i*^*th*^ animal in the *k*^*th*^ population; *a*_*jk*_ is the *j*^*th*^ SNP effect in population *k,* and *e*_*ik*_ is the random residual effect; and in the matrix notation, y_k_, a_k_, and e_k_ are vectors of phenotypic values, SNP effects, and residual effects, respectively; 1 is a vector with all elements set to 1; and X_k_ is the design matrix relating y_k_ to a_k_. In the model, *a*_*jk*_ allows the *j*^*th*^ SNP effect to have a different value in population *k*. To share information among different populations, a common latent indicator variable indicating whether SNP *j* is associated with a QTL is used across populations. Accommodating these features into a Bayesian model produces the multi-task Bayesian learning model.

The following prior distributions for the unknown parameters and hyper-parameters are assumed in the multi-task Bayesian learning model:

$$\mu_{k}\sim \mathrm{flat}\;\mathrm{distribution},$$

$$\left(a_{\mathrm{jk}}|\gamma_{j},\sigma_{\mathrm{ak}}^{2}\right)\sim \gamma_{j}N\left(0,\sigma_{\mathrm{ak}}^{2}\right)+\left(1-\gamma_{j}\right)\delta_{0}\left(a_{\mathrm{jk}}\right),$$

$$\left(\gamma_{j}|w\right)\sim w^{1-\gamma_{j}}{\left(1-w\right)}^{\gamma_{j}},$$

$$w\sim \mathrm{Uniform}\left(0,1\right),$$

$$\left(\sigma_{\mathrm{ak}}^{2}|v_{\mathrm{ak}},s_{\mathrm{ak}}^{2}\right)\sim \frac{{\left(v_{\mathrm{ak}}s_{\mathrm{ak}}^{2}/2\right)}^{v_{\mathrm{ak}}/2}}{\Gamma \left(v_{\mathrm{ak}}/2\right)}{\left(\sigma_{\mathrm{ak}}^{2}\right)}^{-\left(1+v_{\mathrm{ak}}\right)/2}\exp\left(-\frac{v_{\mathrm{ak}}s_{\mathrm{ak}}^{2}}{2\sigma_{\mathrm{ak}}^{2}}\right),$$

$$\left(e_{k}|\sigma_{\mathrm{ek}}^{2}\right)\sim N\left(0,I\sigma_{\mathrm{ek}}^{2}\right),$$

$$\left(\sigma_{\mathrm{ek}}^{2}|v_{\mathrm{ek}},s_{\mathrm{ek}}^{2}\right)\sim \frac{{\left(v_{\mathrm{ek}}s_{\mathrm{ek}}^{2}/2\right)}^{v_{\mathrm{ek}}/2}}{\Gamma \left(v_{\mathrm{ek}}/2\right)}{\left(\sigma_{\mathrm{ek}}^{2}\right)}^{-\left(1+v_{\mathrm{ek}}\right)/2}\exp\left(-\frac{v_{\mathrm{ek}}s_{\mathrm{ek}}^{2}}{2\sigma_{\mathrm{ek}}^{2}}\right).$$

The likelihood function of the whole data given all the parameters in the model is:

$$\prod_{k=1}^{c}{\left(2\pi \sigma_{\mathrm{ek}}^{2}\right)}^{-\frac{n_{k}}{2}}\exp\left[-\frac{1}{2}\sigma_{\mathrm{ek}}^{-2}{\left(y_{k}-\mu_{k}-X_{k}a_{k}\right)}^{'}\left(y_{k}-\mu_{k}-X_{k}a_{k}\right)\right]$$

So the joint posterior density function is:

$$\begin{array}{l} f\left(\sigma_{a}^{2},\sigma_{e}^{2},w,\gamma ,a,\mu |y\right)\propto \prod_{k=1}^{c}\left\{{\left(\sigma_{\mathrm{ek}}^{2}\right)}^{-\frac{v_{\mathrm{ek}}+n_{k}}{2}-1}\exp\left[-\frac{{\left(y_{k}-\mu_{k}-X_{k}a_{k}\right)}^{'}\left(y_{k}-\mu_{k}-X_{k}a_{k}\right)+v_{\mathrm{ek}}s_{\mathrm{ek}}^{2}}{2\sigma_{\mathrm{ek}}^{2}}\right]{\left(\sigma_{\mathrm{ak}}^{2}\right)}^{-\frac{v_{\mathrm{ak}}}{2}-1}\exp\left(-\frac{v_{\mathrm{ak}}s_{\mathrm{ak}}^{2}}{2\sigma_{\mathrm{ak}}^{2}}\right)\right\}\;\prod_{k=1}^{c}\prod_{j=1}^{m}\left\{\left[\gamma_{j}{\left(\sigma_{\mathrm{ak}}^{2}\right)}^{-\frac{1}{2}}\exp\left(-\frac{a_{\mathrm{jk}}^{2}}{2\sigma_{\mathrm{ak}}^{2}}\right)+\left(1-\gamma_{j}\right)\delta_{0}\left(a_{\mathrm{jk}}\right)\right]w^{\left(1-\gamma_{j}\right)}{\left(1-w\right)}^{\gamma_{j}}\right\} \end{array}$$

### Gibbs sampling algorithm

A Gibbs sampling algorithm was designed to draw samples for unknown (hyper-) parameters from their full conditional posterior distributions. To avoid reducibility of Markov chain, γ_*j*_ and *a*_*jk*_ are jointly sampled by first sampling γ_*j*_ from $f\left(\gamma_{j}|\theta_{j^{-}},y\right)$ followed by sampling *a*_*jk*_ from $f\left(a_{\mathrm{jk}}|\gamma_{j},\theta_{j^{-}},y\right),$ where $\theta_{j^{-}}$ represents all parameters except γ_*j*_ and *a*_*jk*_. Full conditional posterior distributions for $\mu_{k},w,\sigma_{\mathrm{ak}}^{2}\;\mathrm{and}\;\sigma_{\mathrm{ek}}^{2}\;$ can be derived by picking up the relevant parts from the joint posterior distribution. Derivation for the density function $f\left(\gamma_{j}|\theta_{j^{-}},y\right)$ and sampling of γ_*j*_ are described in the Appendix. The Gibbs sampling steps are described as below:

Step 1. Initialize the parameters

$$w,\gamma ,\sigma_{\mathrm{ak}}^{2},\sigma_{\mathrm{ek}}^{2},\mu_{k}\;\mathrm{and}\;a_{k}.$$

Step 2. For *j=1,···, m*

a. Sample γ_*j*_ from Bernoulli distribution with probability 1/(1+*q*_*j*_),

$$q_{j}=\frac{w}{1-w}\sqrt{\prod_{k}\left(x_{\mathrm{jk}}^{\prime }x_{\mathrm{jk}}\frac{\sigma_{\mathrm{ak}}^{2}}{\sigma_{\mathrm{ek}}^{2}}+1\right)}\exp\left(-\frac{1}{2}\sum_{k}\frac{{\hat{\mu }}_{a_{\mathrm{jk}}}^{2}}{{\hat{\sigma }}_{a_{\mathrm{jk}}}^{2}}\right)$$

in which

$${\hat{\mu }}_{a_{\mathrm{jk}}}=\frac{x_{\mathrm{jk}}^{'}\left(y_{k}-{\hat{\mu }}_{k}-X_{1k:\mathrm{jk}-1}{\hat{a}}_{1k:\mathrm{jk}-1}^{l+1}-X_{\mathrm{jk}+1:\mathrm{mk}}{\hat{a}}_{\mathrm{jk}+1:\mathrm{mk}}^{l}\right)}{x_{\mathrm{jk}}^{'}x_{\mathrm{jk}}+\sigma_{\mathrm{ek}}^{2}/\sigma_{\mathrm{ak}}^{2}},$$

and

$${\hat{\sigma }}_{a_{\mathrm{jk}}}^{2}=\frac{\sigma_{\mathrm{ek}}^{2}}{x_{\mathrm{jk}}^{'}x_{\mathrm{jk}}+\sigma_{\mathrm{ek}}^{2}/\sigma_{\mathrm{ak}}^{2}}$$

(see Appendix for details).

b. For *k=*1,*···c*, sample *a*_*jk*_ from

$$f\left(a_{\mathrm{jk}}|\gamma_{j},\theta_{j^{-}},y\right)=\left\{\begin{array}{cc} \delta_{0}\left(a_{\mathrm{jk}}\right) & \mathrm{if}\gamma_{j}=0\\ N\left({\hat{\mu }}_{a_{\mathrm{jk}}},{\hat{\sigma }}_{a_{\mathrm{jk}}}^{2}\right) & \mathrm{if}\gamma_{j}=1 \end{array}\right)$$

Step 3. Sample *w* from Beta distribution$f\left(w|\gamma \right)=\frac{1}{B\left(\alpha ,\beta \right)}w^{\alpha -1}{\left(1-w\right)}^{\beta -1},$ where *α* = *m* - ∑ *γ*_*j*_ and *β* = ∑ *γ*_*j*_.

Step 4. For *k=*1,···, *c*, sample $\sigma_{\mathrm{ak}}^{2}$ from scaled inverse Chi-square distribution

$$\chi^{-2}\left(v_{\mathrm{ak}}+\sum \gamma_{j},\frac{v_{\mathrm{ak}}s_{\mathrm{ak}}^{2}+\sum \gamma_{j}a_{\mathrm{jk}}^{2}}{v_{\mathrm{ak}}+\sum \gamma_{j}}\right)$$

Step 5. For *k=*1,···, *c*, sample $\sigma_{\mathrm{ek}}^{2}$ from scaled inverse Chi-square distribution

$$\chi^{-2}\left(v_{\mathrm{ek}}+n_{k},\frac{v_{\mathrm{ek}}s_{\mathrm{ek}}^{2}+{\left(y_{k}-\mu_{k}-X_{k}a_{k}\right)}^{\prime }\left(y_{k}-\mu_{k}-X_{k}a_{k}\right)}{v_{\mathrm{ek}}+n_{k}}\right)$$

Step 6. For *k=*1,···, *c*,

Sample *μ*_*k*_ from $N\left(\frac{{\left(y_{k}-X_{k}{\hat{a}}_{k}\right)}^{'}\left(y_{k}-X_{k}{\hat{a}}_{k}\right)}{n_{k}},\frac{{\hat{\sigma }}_{e_{k}}^{2}}{n_{k}}\right)$ Repeat Step 2 to 6 until a set number of iterations are reached.

It can be shown in step 2 that information from all populations are used to generate the latent variable γ_*j*_, and the SNP effect *a*_*jk*_ is generated for each population.

### Computer program

Computer programs were written in C language to implement the multi-task Bayesian learning model, single-task Bayesian learning and the simple data pooling method. Programs and source codes are available upon request.

### Monte Carlo simulations

The aim of the simulation was to evaluate the performance of the proposed multi-task Bayesian learning model and to compare with the single-task model and the simple data pooling method. Different scenarios were considered that differ in number of QTL affecting the trait, correlations of the QTL effects between different populations, and the density of SNP panels used for genomic prediction.

Real genotypes from 458 Ayrshire and 2,298 Holstein bulls were used for simulations. All Ayrshire animals and 690 Holstein animals were genotyped on the Illumina BovineHD BeadChip (800 k) SNP panel, and the remaining 1,608 Holstein animals were genotyped on the Illumina BovineSNP50 BeadChip (50 k) SNP panel. SNPs meeting one of the following criteria were excluded: minor allele frequency (MAF) lower than 0.05, missing genotype rate greater than 0.10, highly correlated with any other SNP genotype (95% genotypes from two loci identical or in complementary). SNP locations were determined against Bovine genome assembly UMD3.1 [22]. SNPs with unknown locations or on sex chromosomes were discarded. SNPs filtered in one breed were also removed from the other breed. After editing, 246,668 SNPs from the 800 k panel and 28,206 SNPs from the 50 k panel were kept for analyses.

For ease of computation, only SNPs from the first 10 chromosomes were used. Four scenarios were considered with combinations of QTL number being either 20 or 200 and the correlation of QTL effects between the two populations being low (ρ = 0.2) or high (ρ = 0.8). For each scenario, QTL were randomly sampled from the 50 k panel. For each QTL *j*, allele substitution effects in the two populations, α_*j*1_ and α_*j*2_, were sampled from a bivariate normal distribution with mean 0 and variance-covariance structure $\sum =\left(\begin{array}{cc} 1 & \rho \\ \rho & 1 \end{array}\right)\sigma^{2}$ in which *σ*^2^ = 1. Breeding value of each animal *i*, was calculated as $a_{i}=\sum_{j}X_{j}\alpha_{j}$ where *X*_*j*_ is the QTL genotypes coded as 0, 1 or 2, number of copies on an arbitrarily chosen allele. Total additive genetic variance were calculated within each breed as $\sigma_{a}^{2}=\sum_{j}2p_{j}\left(1-p_{j}\right)\alpha_{j}^{2}$, where *p*_*j*_ is the QTL allele frequency. For each animal *i*, a residual effect *e*_*i*_, was sampled from a normal distribution with mean 0 and variance $\sigma_{e}^{2}$, so that phenotypic value of the animal *y*_*i*_*=a*_*i*_*+e*_*i*_. $\sigma_{e}^{2}$ was equal to $\sigma_{a}^{2}$ to give a trait with heritability of 0.5. Each scenario was replicated 10 times.

Four SNP panels of different density, the original 800 k panel, and the mimicked 400 k, 200 k, 100 k by selecting every 2nd, 4th, and 8th SNP, respectively, from the 800 k panel were used for genomic prediction. For each scenario described above, the simulated QTL were removed from the SNP panels. A special scenario was designed to keep all QTL genotypes in the 800 k panel for genomic prediction. The 50 k genotypes of 1,608 Holstein animals were imputed to 800 k using genotype imputation software FImpute developed by Sargolzaei et al. [23]. Imputation accuracy was evaluated on a set of 126 animals that have been genotyped on both the 50 k and 800 k panel, and the ratio of the genotypes that were correctly imputed was 0.9930.

For training and validation purposes, 393 Ayrshire and 2,084 Holstein animals born before 2004 were used as training set, 65 Ayrshire and 214 Holstein animals born in and after 2004 were used for validation. The number of animals used for genomic prediction is shown in Table 1. The simulated phenotypic values for training animals were used to derive SNP effects using different models. Degree of freedom of the inverse chi-square distributions for variances of SNP effects and residual effects were set to 4 and 10, respectively. The scale parameter $S_{a}^{2}$ was derived from the expected value of a scaled inverse chi-square distributed random variable, i.e., *E*(*σ*^2^) = *S*^2^*v*/(*v* - 2) and hence $S_{a}^{2}=\sigma_{a}^{2}\left(v_{a}-2\right)/v_{a}$ where $\sigma_{a}^{2}$ is the true additive genetic variance. $S_{e}^{2}$ was derived similarly. The Gibbs chain was run for 50,000 cycles with the first 10,000 discarded as burn-in. Marker effects were estimated as averages from all post burn-in samples. Genomic breeding values for animals in the validation set were estimated as the sum of population mean and all marker effects. Accuracy was evaluated as Pearson’s correlation coefficient between genomic estimated breeding values and true breeding values for validation animals, i.e. r(GEBV, TBV). Regression of true breeding value on genomic estimated breeding values, b(TBV, GEBV), was also calculated to evaluate the bias of the genomic estimated breeding values.

**Table 1:** Number of animals used for genomic prediction

	**Ayrshire**	**Holstein**
Training set	393	2084
Validation set	65	214
Total	458	2298

### Real data

Five milk production traits including milk yield, fat yield, protein yield, fat percentage and protein percentage were used for the same set of animals in the simulation study. Bull proofs (estimated breeding values from progeny testing, EBV) from April 2008 were used as phenotypes for training animals, and bull proofs from December 2011 were used for validation animals. All proofs were provided by Canadian Dairy Network (CDN) and had reliability above 0.65.

Two SNP panels with 28,206 SNPs from the 50 k panel and 246,668 SNPs from the 800 k panel were used for genomic prediction. Degree of freedom of the inverse chi-square distributions for variances of SNP effects and residual effects were set to 4 and 10, respectively. Scale parameters for the two distributions were derived in the same way as described in the simulation study, but instead of using true additive genetic variance and residual variance, estimated variances were used. Estimated additive genetic variances and residual variance were obtained from ASReml [24] by fitting an animal model with a population mean, animal effect, and random residual effect. The pedigree contained 15,731 animals for the Holstein population and 4,926 animals for Ayrshire. For 28,206 SNPs, the Gibbs sampling procedure was run for 50,000 iterations with the first 10,000 discarded as burn-in; and for 246,668 SNPs, the Gibbs sampling procedure was run for 100,000 iterations with the first 50,000 discarded as burn-in. Burn-in period was determined by visually inspecting the Gibbs chain. All samples after the burn-in period were kept. SNP effects were estimated by averaging all samples after the burn-in period. After the estimation of SNP effects, the GEBV was calculated for animals in the validation set by summing up the population mean and all the SNP effects. Accuracy was measured as Pearson’s correlation coefficient between GEBV and the 2011 bull proofs for validation animals.

## Results

### Monte Carlo simulation study

Table 2 shows the accuracy of genomic prediction with simulated 20 QTL. In the Ayrshire validation set, multi-task Bayesian learning model performed the best among the three methods within each SNP panel used under the scenario with either a low (ρ = 0.2) or high (ρ = 0.8) correlation of simulated QTL effects between Ayrshire and Holstein populations. The greatest increase of accuracy was observed when ρ was 0.8 and when density of the SNP panel was the highest (accuracy increased from 0.67 for the single-task method to 0.83 for the multi-task method). Simply pooling data together substantially reduced the prediction accuracy in Ayshire when ρ was 0.2. The greatest reduction of accuracy was observed when ρ was 0.2 and when density of the SNP panel was the highest (accuracy decreased from 0.71 for the single-task method to 0.56 for the pooling method). When ρ was 0.8, the pooling method had an increased accuracy in Ayrshire compared with the single-task method. The pooling method produced substantially lower accuracy than the multi-task model in the Ayrshire validation set, especially when QTL effects were lower correlated between the two populations. In the Holstein validation set, the multi-task model performed similar or slightly better than the single-task model. The pooling method had a slightly reduction of accuracy when ρ was 0.2, and had a similar accuracy compared with the single-task method when ρ was 0.8. Within each method, increasing density of the SNP panel generally improved prediction accuracy in both the Ayrshire and Holstein populations. Table 2 also shows the slopes of regression of true breeding values on the GEBV. Regression coefficients of true breeding values on GEBV were less than one for the pooling method indicating that the GEBV were inflated.

**Table 2:** Accuracy {expressed as correlations between true breeding values (TBV) and genomic estimated breeding values [GEBV; r(TBV, GEBV)]}, and slopes [b(TBV, GEBV)] of regression of TBV on GEBV for genomic prediction with 20 simulated QTL

	**Ayrshire**			**Holstein**		
**SNP panel**	**Single-task**	**Pooling**	**Multi-task**	**Single-task**	**Pooling**	**Multi-task**
r(TBV, GEBV)						
ρ = 0.2						
800 k	0.71 ± 0.02	0.56 ± 0.04	0.75 ± 0.02	0.91 ± 0.01	0.90 ± 0.01	0.91 ± 0.01
400 k	0.64 ± 0.04	0.53 ± 0.04	0.73 ± 0.03	0.90 ± 0.01	0.86 ± 0.01	0.90 ± 0.01
200 k	0.60 ± 0.05	0.50 ± 0.04	0.68 ± 0.02	0.88 ± 0.01	0.84 ± 0.01	0.88 ± 0.01
100 k	0.57 ± 0.04	0.47 ± 0.04	0.63 ± 0.03	0.84 ± 0.01	0.81 ± 0.02	0.84 ± 0.01
ρ = 0.8						
800 k	0.67 ± 0.05	0.76 ± 0.02	0.83 ± 0.01	0.92 ± 0.01	0.92 ± 0.01	0.93 ± 0.01
400 k	0.66 ± 0.05	0.72 ± 0.02	0.80 ± 0.01	0.90 ± 0.01	0.89 ± 0.01	0.90 ± 0.01
200 k	0.66 ± 0.04	0.68 ± 0.02	0.76 ± 0.01	0.86 ± 0.01	0.86 ± 0.01	0.86 ± 0.01
100 k	0.61 ± 0.04	0.63 ± 0.04	0.72 ± 0.02	0.83 ± 0.01	0.83 ± 0.01	0.84 ± 0.01
b(TBV, GEBV)						
ρ = 0.2						
800 k	1.06 ± 0.05	0.73 ± 0.05	1.04 ± 0.05	0.98 ± 0.02	1.01 ± 0.01	0.98 ± 0.01
400 k	1.06 ± 0.05	0.76 ± 0.06	1.06 ± 0.05	0.99 ± 0.02	1.00 ± 0.01	0.98 ± 0.01
200 k	1.02 ± 0.06	0.75 ± 0.05	1.03 ± 0.04	1.00 ± 0.01	0.99 ± 0.01	0.98 ± 0.01
100 k	1.00 ± 0.06	0.75 ± 0.05	1.03 ± 0.06	1.00 ± 0.01	1.00 ± 0.02	0.99 ± 0.01
ρ = 0.8						
800 k	1.10 ± 0.06	0.90 ± 0.03	1.10 ± 0.04	0.99 ± 0.02	1.00 ± 0.02	0.99 ± 0.02
400 k	1.09 ± 0.06	0.89 ± 0.04	1.07 ± 0.04	0.99 ± 0.02	0.99 ± 0.01	0.99 ± 0.02
200 k	1.14 ± 0.06	0.89 ± 0.06	1.04 ± 0.05	0.98 ± 0.02	1.00 ± 0.02	0.98 ± 0.03
100 k	1.08 ± 0.08	0.85 ± 0.06	1.06 ± 0.05	0.98 ± 0.03	0.99 ± 0.03	0.98 ± 0.03

Table 3 shows the accuracy of genomic prediction with simulated 200 QTL. In the Ayrshire validation set, the multi-task model had slightly higher prediction accuracy within each SNP panel compared with the single-task model under either scenario with low (ρ =0.2) or high (ρ =0.8) correlated QTL effects between the Ayrshire and Holstein populations. Pooling method performed similar or slightly worse compared with single-task model when ρ was 0.2, and generally performed better when ρ was 0.8. The pooling method also performed slightly better than the multi-task model when ρ was 0.8 and when density of the SNP panel was relatively high. When ρ was 0.2, regression coefficients of true breeding values on GEBV were less than one for the pooling method indicating that the GEBV were inflated. The three methods performed similar in the Holstein validation set. Overall, the accuracy was lower compared with scenarios when only 20 QTL were simulated regardless of the methods used.

**Table 3:** Accuracy {expressed as correlations between true breeding values (TBV) and genomic estimated breeding values [GEBV; r(TBV, GEBV)]}, and slopes [b(TBV, GEBV)] of regression of TBV on GEBV for genomic prediction with 200 simulated QTL

	**Ayrshire**			**Holstein**		
**SNP panel**	**Single-task**	**Pooling**	**Multi-task**	**Single-task**	**Pooling**	**Multi-task**
r(TBV, GEBV)						
ρ = 0.2						
800 k	0.46 ± 0.02	0.44 ± 0.04	0.47 ± 0.03	0.77 ± 0.01	0.76 ± 0.01	0.77 ± 0.01
400 k	0.46 ± 0.02	0.43 ± 0.03	0.47 ± 0.02	0.76 ± 0.01	0.76 ± 0.01	0.76 ± 0.01
200 k	0.46 ± 0.02	0.42 ± 0.04	0.47 ± 0.02	0.75 ± 0.01	0.75 ± 0.01	0.75 ± 0.01
100 k	0.45 ± 0.02	0.41 ± 0.03	0.46 ± 0.03	0.74 ± 0.01	0.74 ± 0.01	0.74 ± 0.01
ρ = 0.8						
800 k	0.54 ± 0.04	0.57 ± 0.02	0.56 ± 0.03	0.74 ± 0.02	0.75 ± 0.01	0.75 ± 0.02
400 k	0.54 ± 0.03	0.56 ± 0.03	0.55 ± 0.03	0.74 ± 0.02	0.74 ± 0.02	0.74 ± 0.02
200 k	0.54 ± 0.03	0.56 ± 0.02	0.55 ± 0.03	0.73 ± 0.02	0.73 ± 0.02	0.73 ± 0.02
100 k	0.53 ± 0.03	0.52 ± 0.02	0.54 ± 0.03	0.72 ± 0.02	0.72 ± 0.02	0.72 ± 0.02
b(TBV, GEBV)						
ρ = 0.2						
800 k	1.14 ± 0.08	0.89 ± 0.09	1.16 ± 0.09	1.07 ± 0.01	1.09 ± 0.01	1.07 ± 0.01
400 k	1.14 ± 0.09	0.91 ± 0.08	1.13 ± 0.09	1.07 ± 0.01	1.09 ± 0.01	1.07 ± 0.01
200 k	1.14 ± 0.09	0.88 ± 0.08	1.14 ± 0.09	1.08 ± 0.01	1.09 ± 0.01	1.08 ± 0.01
100 k	1.15 ± 0.09	0.89 ± 0.09	1.15 ± 0.09	1.09 ± 0.02	1.11 ± 0.03	1.09 ± 0.02
ρ = 0.8						
800 k	1.12 ± 0.11	1.00 ± 0.06	1.07 ± 0.09	1.01 ± 0.02	1.00 ± 0.02	1.01 ± 0.02
400 k	1.11 ± 0.11	1.02 ± 0.07	1.08 ± 0.09	1.01 ± 0.02	1.00 ± 0.02	1.01 ± 0.02
200 k	1.12 ± 0.11	1.04 ± 0.07	1.11 ± 0.09	1.01 ± 0.02	1.01 ± 0.02	1.01 ± 0.02
100 k	1.11 ± 0.11	0.99 ± 0.07	1.10 ± 0.10	1.01 ± 0.02	1.01 ± 0.02	1.01 ± 0.02

To evaluate the performance of the three methods under situations where all QTL genotypes can be acquired and included for genomic prediction, Table 4 shows the accuracy of genomic prediction when QTL genotypes are included together with SNP marker genotypes for training and validation. When 20 QTL were simulated, using multi-task model improved the accuracy by 0.16 and 0.22 in the Ayrshire validation set for scenarios where correlation of QTL effects between the Ayrshire and Holstein populations was 0.2 and 0.8, respectively. Using the pooling method reduced the accuracy in Ayrshire by 0.12 when ρ was 0.2, but increased the accuracy by 0.17 when ρ was 0.8. When 200 QTL were simulated, using the multi-task model increased the prediction accuracy by 0.03 and 0.07, respectively, for ρ equal to 0.2 and 0.8. The pooling method reduced the accuracy by 0.04 when ρ was 0.2, and increased the accuracy by 0.11 when ρ was 0.8. The pooling method outperformed the multi-task model only for the scenario where 200 QTL were simulated with their effects highly correlated between the two populations. In the Holstein validation set, the multi-task model had similar or slightly higher accuracy compared with the single-task model. The pooling method had similar accuracy as the single-task model when ρ was 0.8. When ρ was 0.2, the pooling method had slightly lower accuracy compared with the single-task model. Regression coefficients of true breeding values on GEBV were less than one for the pooling method except for the scenario of 200 simulated QTL with effects highly correlated between the two populations, indicating that the GEBV predicted by the pooling method were inflated.

**Table 4:** Accuracy {expressed as correlations between true breeding values (TBV) and genomic estimated breeding values [GEBV; r(TBV, GEBV)]}, and slopes [b(TBV, GEBV)] of regression of TBV on GEBV for genomic prediction with simulated QTL genotypes included for training and validation

		**Ayrshire**			**Holstein**		
**No. of QTL**	**ρ**	**Single-task**	**Pooling**	**Multi-task**	**Single-task**	**Pooling**	**Multi-task**
r(TBV, GEBV)							
20	0.2	0.76 ± 0.04	0.64 ± 0.04	0.92 ± 0.01	0.96 ± 0.01	0.93 ± 0.01	0.97 ± 0.01
20	0.8	0.71 ± 0.05	0.88 ± 0.02	0.93 ± 0.01	0.97 ± 0.01	0.97 ± 0.01	0.97 ± 0.01
200	0.2	0.57 ± 0.03	0.53 ± 0.03	0.60 ± 0.04	0.77 ± 0.01	0.76 ± 0.01	0.78 ± 0.01
200	0.8	0.54 ± 0.04	0.65 ± 0.02	0.61 ± 0.04	0.76 ± 0.02	0.78 ± 0.02	0.77 ± 0.01
b(TBV, GEBV)							
20	0.2	1.01 ± 0.06	0.83 ± 0.08	0.99 ± 0.03	1.00 ± 0.01	1.00 ± 0.02	1.00 ± 0.01
20	0.8	1.04 ± 0.08	0.89 ± 0.06	0.97 ± 0.03	0.98 ± 0.01	1.01 ± 0.02	0.99 ± 0.01
200	0.2	1.23 ± 0.10	0.89 ± 0.06	1.16 ± 0.08	1.00 ± 0.03	1.03 ± 0.03	1.01 ± 0.03
200	0.8	1.10 ± 0.09	1.06 ± 0.06	1.12 ± 0.09	0.98 ± 0.03	0.97 ± 0.03	0.98 ± 0.02

### Real data analysis

Table 5 shows the accuracy of genomic prediction for milk production traits using real data. For the Ayrshire validation set, the multi-task model increased the accuracy by up to 0.07 compared with the single-task model, while the simple data pooling method resulted in reduced accuracy in general. The greatest increase in accuracy using multi-task model compared with single-task model was for fat percentage (0.06) and protein percentage (0.07) when 28,206 SNPs were used. For the Holstein validation set, the single-task model, simple data pooling method, and the multi-task model performed similar regardless of the traits studied.

**Table 5:** Accuracy of genomic prediction of breeding values for milk production traits

	**Ayrshire**			**Holstein**		
**Trait**	**Single-task**	**Pooling**	**Multi-task**	**Single-task**	**Pooling**	**Multi-task**
No. of SNP: 28,206						
Milk yield	0.52	0.44	0.54	0.66	0.65	0.66
Fat yield	0.64	0.55	0.66	0.63	0.64	0.63
Protein yield	0.70	0.60	0.70	0.69	0.69	0.68
Fat %	0.66	0.58	0.72	0.74	0.74	0.74
Protein %	0.48	0.51	0.55	0.67	0.68	0.67
No. of SNP: 246,668						
Milk yield	0.54	0.53	0.55	0.64	0.64	0.64
Fat yield	0.67	0.62	0.67	0.63	0.64	0.63
Protein yield	0.72	0.68	0.72	0.66	0.66	0.66
Fat %	0.66	0.65	0.69	0.77	0.77	0.78
Protein %	0.51	0.42	0.53	0.71	0.68	0.70

## Discussion

Traditionally, genomic prediction with data from multiple populations were implemented either by running genomic prediction within each population (single-task) or by simply pooling data together. Single-task genomic prediction cannot utilize information from other populations and therefore, the accuracy of genomic prediction is largely determined by the size of training data set [2,3]. For breeds with only a small number of animals having both DNA marker genotypes and phenotype data, the accuracy of genomic prediction can be low [25]. Combining the data with other breed populations has the potential to improve the prediction accuracy. It is however, difficult to effectively account for the differences of SNP effects among different populations by simply pooling data together. If the marker density is low or the populations are divergent from each other, simply pooling data together may result in unfavorable prediction accuracies [6]. The multi-task Bayesian learning model proposed in this study uses information from all populations simultaneously while allowing the SNP effects to vary in different populations. Different populations share information through a common set of latent indicator variables. When the target trait has a similar genetic background in related populations, it is reasonable to assume that some shared QTL affecting a common trait in different populations. However, the linkage disequilibrium phase between SNP markers and QTL are likely to be inconsistent, especially when the marker density is low. Therefore, the multi-task Bayesian learning model is more flexible about the SNP effects and is likely to have better performance than a simple data pooling method.

Results from simulation studies support the use of multi-task Bayesian model for multi-population genomic prediction especially when there are a few QTL affecting the trait. For the scenario where a few QTL affect the trait, the increase of accuracy by using multi-task model was greater when QTL effects had a higher correlation between two populations. The accuracy was further increased when QTL genotypes were included for training and validation. These results are expected as the higher the correlation of QTL effects between two populations, the more informative information the two populations can share. Including QTL genotypes also increased the amount of information to be shared between the two populations. Results suggest that the proposed multi-task Bayesian learning model is effective in combining information from multi-populations to improve accuracy of genomic prediction.

Results from simulation also showed that simply pooling data together may reduce the accuracy of genomic prediction if QTL effects were lowly correlated between two populations or if a relatively low density SNP panel was used. When QTL effects had a high correlation between two populations and the SNP panel was high, the pooling method increased the accuracy compared with single-task model. When the correlation of QTL effects between two populations was high, the pooling method was inferior to the multi-task model if the number of QTL was small, but outperformed the multi-task model if the number of QTL was large. These results are reasonable since the pooling method assumes the same SNP effects among different populations. This assumption will be violated if the correlation of QTL effects is low between two populations and may result in poor performance of the pooling method. The multi-task model proposes to share information through the same latent indicator variables. Correlations of QTL effects among different populations are not explicitly used and therefore, information across populations may not be fully utilized if such correlations are high. This might be why the pooling method still outperformed the multi-task model when the number of QTL is large and the correlation of QTL effects between two populations was high.

Results from real data analyses in this study showed that the multi-task Bayesian learning model produced a similar or higher accuracy compared to the single-task model, and that simply pooling data together resulted in a reduced accuracy when the marker density was low. SNP effects could be different across populations, especially when lower density markers were used [6]. These results are in agreement with results from simulation studies.

Gains of accuracy by using the multi-task model were higher for fat percentage and protein percentage traits than for other traits. This is likely due to that large QTL or genes such as DGAT1, have larger influence on the percentage traits than on the yield traits [26,27]. These results agreed with simulation studies which showed that the multi-task model performed better when there are fewer QTL affecting the trait.

In this study, only two dairy cattle breeds were considered, and the multi-task Bayesian learning model was shown to be effective and more beneficial to the population with a smaller data size. For the larger population, using multi-task model did not produce much improvement. The little improvement in the larger population could be due to that the smaller population is too small to be able to have a significant impact on the larger population. In practice, there are situations where many populations are to be combined for analysis with each one contributing a small amount of data, a typical example as in beef cattle production. It would be interesting to test the performance of the multi-task model under such scenarios.

The current multi-task model considered additive genetic effects only. Non-additive genetic effects, which have gained growing interests with availability of genomics information [28-30], can also be accommodated into the multi-task model. A similar strategy used in this study to sharing information across populations for additive genetic effects may also be applied to non-additive genetic effects. Inclusion of non-additive genetic effects in the multi-task model warrants further investigation.

The proposed multi-task Bayesian learning model used a spike and slab mixture distribution to conduct variable selection and shrinkage for SNP effects. This prior setting is similar to that in the BayesCπ method proposed by Habier et al. [21]. Other types of mixture distributions currently being used for genomic prediction [1,19,20], can also be adapted to the multi-task model. The strategy used in the multi-task Bayesian learning model allows different populations to share information through a common latent variable assumed for each SNP. Such strategy has been shown effective in both simulation and real data studies; however, other strategies may also be exploited, for example, by modeling the joint distribution of the SNP effects among different populations. Further investigations are required to evaluate alternative strategies for sharing information across populations.

In this study, the Bayesian models were implemented via a Gibbs sampling algorithm adopting the residual-update computing strategy proposed by Legarra and Misztal [31]. Computing time for this algorithm is proportional to the number of animals and number of SNPs in the model. For the training sample size of 2,477 animals and genotypes of 246,668 SNPs, the proposed multi-task model requires 44 CPU hours to complete 150,000 Gibbs sampling cycles on a Linux cluster system with Intel X5675 3.07GHz CPU. With increased training sample size and increasing marker density to a very high density panel or even sequence data, computing burdens would become a concern. A recent study [32] has improved the residual-update algorithm resulting in the CPU time reduced by 35.3 to 43.3%. The authors in the same study [32] also proposed an alternative algorithm which reduced CPU time by 74.5 to 93.0%. Approximation algorithms based on expectation-maximization (EM) [33-35] and variational Bayes [36] have also been proposed to replace the time consuming Markov chain Monte Carlo (MCMC) sampling based algorithms. Adapting these algorithms to accommodate the multi-task Bayesian learning model would be of great interest.

## Conclusions

A multi-task Bayesian learning model was proposed for multi-population genomic prediction. The multi-task model shares information across populations through a common set of latent indicator variables while allowing the SNP effects to vary in different populations. Simulation studies and real data analysis suggest that the proposed multi-task Bayesian learning model is effective and beneficial to populations where a small number of training animals are available. Accuracy of genomic prediction in small populations can be improved by using the multi-task model especially for traits affected by a few QTL with large effects.

## Appendix

**Sampling of *r***_***j ***_**in the multi-task Bayesian learning model**

With the assumption of independence between $\theta_{j^{-}}$ and *r*_*j*_, by Bayes’ theorem, one has

(A.1) $$\begin{array}{ll} f\left(r_{j}=1|\theta_{j^{-}},y\right) & =\frac{f\left(y|r_{j}=1,\theta_{j^{-}}\right)f\left(r_{j}=1\right)}{f\left(y|r_{j}=1,\theta_{j^{-}}\right)f\left(r_{j}=1\right)+f\left(y|r_{j}=0,\theta_{j^{-}}\right)f\left(r_{j}=0\right)}\\ & =\frac{1}{1+q_{j}}, \end{array}$$

where

(A.2) $$\begin{array}{ll} \;q_{j} & =\frac{f\left(y|r_{j}=0,\theta_{j^{-}}\right)f\left(r_{j}=0\right)}{f\left(y|r_{j}=1,\theta_{j^{-}}\right)f\left(r_{j}=1\right)}\\ & =\frac{f\left(r_{j}=0\right)}{f\left(r_{j}=1\right)}\prod_{k=1}^{c}\frac{f\left(y_{k}|r_{j}=0,\theta_{j^{-}k}\right)}{f\left(y_{k}|r_{j}=1,\theta_{j^{-}k}\right)}. \end{array}$$

Similarly,

$$f\left(r_{j}=0|\theta_{j^{-}},y\right)=\frac{q_{j}}{1+q_{j}}.$$

Next, the conditional likelihoods $f\left(y_{k}|r_{j}=0,\theta_{j^{-}k}\right)$ and $f\left(y_{k}|r_{j}=1,\theta_{j^{-}k}\right)$ will be derived.

Suppose one is at the (*l*+1)^*th*^ Gibbs sampling iteration, and wants to sample *γ*_*j*_ and *a*_*jk*_, the linear regression model given all parameters except *γ*_*j*_ and *a*_*jk*_ can be written as:

$$y_{k}^{*}=x_{\mathrm{jk}}a_{\mathrm{jk}}+e_{k},$$

Where

$$y_{k}^{*}=y_{k}-{\hat{\mu }}_{k}-X_{1k:\mathrm{jk}-1}{\hat{a}}_{1k:\mathrm{jk}-1}^{l+1}-X_{\mathrm{jk}+1:\mathrm{mk}}{\hat{a}}_{\mathrm{jk}+1:\mathrm{mk}}^{l}.$$

Given the priors that $\left(a_{\mathrm{jk}}|\gamma_{j}\right)\sim \gamma_{j}N\left(0,\sigma_{\mathrm{ak}}^{2}\right)+\left(1-\gamma_{j}\right)\delta_{0}\left(a_{\mathrm{jk}}\right)$ and $\left(e_{k}|\sigma_{\mathrm{ek}}^{2}\right)\sim N\left(0,I\sigma_{\mathrm{ek}}^{2}\right),$ the conditional likelihood of **y**_**k**_ can be written as:

(A.3) $$f\left(y_{k}|r_{j}=0,\theta_{j^{-}k}\right)={\left(2\pi \sigma_{\mathrm{ek}}^{2}\right)}^{-n_{k}/2}\exp\left(-\frac{{y_{k}^{*}}^{\prime }y_{k}^{*}}{2\sigma_{\mathrm{ek}}^{2}}\right)$$

And

(A.4) $$f\left(y_{k}|r_{j}=1,\theta_{j^{-}k}\right)={\left(2\pi \right)}^{-n_{k}/2}{\left|V_{y_{k}^{*}}\right|}^{-1/2}\exp\left(-\frac{y_{k}^{*'}V_{y_{k}^{*}}^{-1}y^{*}}{2}\right)$$

Where $V_{y_{k}^{*}}=x_{\mathrm{jk}}x_{\mathrm{jk}}^{'}\sigma_{\mathrm{ak}}^{2}+I\sigma_{\mathrm{ek}}^{2}$ is the (co-) variance matrix of $y_{k}^{*}$ given that *r*_*j*_=1.

Then, $\left|V_{y_{k}^{*}}\right|$ and $V_{y_{k}^{*}}^{-1}$ are derived.

$$\left|V_{y_{k}^{*}}\right|=\left|x_{\mathrm{jk}}x_{\mathrm{jk}}^{'}\sigma_{\mathrm{ak}}^{2}+I\sigma_{\mathrm{ek}}^{2}\right|=\sigma_{\mathrm{ek}}^{2n_{k}}\left|x_{\mathrm{jk}}\frac{\sigma_{\mathrm{ak}}}{\sigma_{\mathrm{ek}}}x_{\mathrm{jk}}^{'}\frac{\sigma_{\mathrm{ak}}}{\sigma_{\mathrm{ek}}}+I\right|,$$

By applying Sylvester’s determinant theorem, one has

(A.5) $$\left|V_{y_{k}^{*}}\right|=\sigma_{\mathrm{ek}}^{2n_{k}}\left(x_{\mathrm{jk}}^{\prime }x_{\mathrm{jk}}\frac{\sigma_{\mathrm{ak}}^{2}}{\sigma_{\mathrm{ek}}^{2}}+1\right)$$

It can be easily verify that $V_{y^{*}}^{-1}$ is:

(A.6) $$V_{y_{k}^{*}}^{-1}=\sigma_{\mathrm{ek}}^{-2}\left(I-\frac{x_{\mathrm{jk}}x_{\mathrm{jk}}^{\prime }}{x_{\mathrm{jk}}^{\prime }x_{\mathrm{jk}}+\sigma_{\mathrm{ek}}^{2}/\sigma_{\mathrm{ak}}^{2}}\right)$$

Substituting A.5 and A.6 into A.4, one has

(A.7) $$\begin{array}{l} f\left(y|r_{j}=1,\theta_{j^{-}}\right)={\left(2\pi \sigma_{\mathrm{ek}}^{2}\right)}^{-n_{k}/2}{\left(x_{\mathrm{jk}}^{\prime }x_{\mathrm{jk}}\frac{\sigma_{\mathrm{ak}}^{2}}{\sigma_{\mathrm{ek}}^{2}}+1\right)}^{-1/2}\\ \;\exp\left\{-\frac{y_{k}^{*'}y_{k}^{*}-{\left(x_{\mathrm{jk}}^{\prime }y_{k}^{*}\right)}^{2}/\left(x_{\mathrm{jk}}^{\prime }x_{\mathrm{jk}}+\sigma_{\mathrm{ek}}^{2}/\sigma_{\mathrm{ak}}^{2}\right)}{2\sigma_{\mathrm{ek}}^{2}}\right\} \end{array}$$

Denote ${\hat{\mu }}_{a_{\mathrm{jk}}}=\frac{x_{\mathrm{jk}}^{'}\left(y_{k}-X_{1k:\mathrm{jk}-1}{\hat{a}}_{1k:\mathrm{jk}-1}^{l+1}-X_{\mathrm{jk}+1:\mathrm{mk}}{\hat{a}}_{\mathrm{jk}+1:\mathrm{mk}}^{l}\right)}{x_{\mathrm{jk}}^{'}x_{\mathrm{jk}}+\sigma_{\mathrm{ek}}^{2}/\sigma_{\mathrm{ak}}^{2}},$${\hat{\sigma }}_{a_{\mathrm{jk}}}^{2}=\frac{\sigma_{\mathrm{ek}}^{2}}{x_{\mathrm{jk}}^{'}x_{\mathrm{jk}}+\sigma_{\mathrm{ek}}^{2}/\sigma_{\mathrm{ak}}^{2}},$ and substitute (A.7) and (A.3) into (A.2), one gets

(A.8) $$q_{j}=\frac{w}{1-w}\sqrt{\prod_{k}\left(x_{\mathrm{jk}}^{\prime }x_{\mathrm{jk}}\frac{\sigma_{\mathrm{ak}}^{2}}{\sigma_{\mathrm{ek}}^{2}}+1\right)}\exp\left(-\frac{1}{2}\sum_{k}\frac{{\hat{\mu }}_{a_{\mathrm{jk}}}^{2}}{{\hat{\sigma }}_{a_{\mathrm{jk}}}^{2}}\right)$$

Finally, *γ*_*j*_ can be drawn from a Bernoulli distribution with probability 1/(1+q_*j*_).

## Competing interests

The authors declare that they have no competing interests.

## Authors’ contributions

LC, CL and FS contributed to the design of the study. LC, CL, SM and FS contributed to the development of the methods and discussions of analysis and results. LC performed the data analysis and drafted the manuscript. All authors read, provided comments and agreed on the contents of the manuscript.

## References

[B1] Meuwissen TH Hayes BJ Goddard ME Prediction of total genetic value using genome-wide dense marker maps Genetics 2001 157 4 1819 1829 11290733 10.1093/genetics/157.4.1819 PMC1461589

[B2] Goddard M Genomic selection: prediction of accuracy and maximisation of long term response Genetica 2009 136 2 245 257 10.1007/s10709-008-9308-0 18704696

[B3] Hayes BJ Goddard ME Technical note: prediction of breeding values using marker-derived relationship matrices J Anim Sci 2008 86 9 2089 2092 10.2527/jas.2007-0733 18407982

[B4] Hayes BJ Bowman PJ Chamberlain AJ Goddard ME Invited review: genomic selection in dairy cattle: progress and challenges (vol 92, pg 433, 2009) J Dairy Sci 2009 92 3 1313 1313 10.3168/jds.2008-1646 19164653

[B5] VanRaden PM Van Tassell CP Wiggans GR Sonstegard TS Schnabel RD Taylor JF Schenkel FS Invited review: Reliability of genomic predictions for north american holstein bulls J Dairy Sci 2009 92 1 16 24 10.3168/jds.2008-1514 19109259

[B6] de Roos APW Hayes BJ Goddard ME Reliability of genomic predictions across multiple populations Genetics 2009 183 4 1545 1553 10.1534/genetics.109.104935 19822733 PMC2787438

[B7] Hayes B Bowman P Chamberlain A Verbyla K Goddard M Accuracy of genomic breeding values in multi-breed dairy cattle populations Genet Sel Evol 2009 41 1 51 19930712 10.1186/1297-9686-41-51 PMC2791750

[B8] Pryce JE Gredler B Bolormaa S Bowman PJ Egger-Danner C Fuerst C Emmerling R Solkner J Goddard ME Hayes BJ Short communication: genomic selection using a multi-breed, across-country reference population J Dairy Sci 2011 94 5 2625 2630 10.3168/jds.2010-3719 21524555

[B9] Erbe M Hayes BJ Matukumalli LK Goswami S Bowman PJ Reich CM Mason BA Goddard ME Improving accuracy of genomic predictions within and between dairy cattle breeds with imputed high-density single nucleotide polymorphism panels J Dairy Sci 2012 95 7 4114 4129 10.3168/jds.2011-5019 22720968

[B10] Brondum RF Su GS Lund MS Bowman PJ Goddard ME Hayes BJ Genome position specific priors for genomic prediction BMC Genomics 2012 13 1 543 10.1186/1471-2164-13-543 23050763 PMC3534589

[B11] Caruana R Multitask learning Mach Learn 1997 28 1 41 75

[B12] Li X Bilmes J Regularized adaptation of discriminative classifiers Proceedings of the 2006 IEEE International Conference on Acoustics, Speech and Signal Processing (ICASSP): 14-19 May 2006; Toulouse 237 240 IEEE 2006(vol. 1)

[B13] Lu Y Lu F Sehgal S Gupta S Du J Tham CH Green P Wan V Cassidy S, Cox F, Mannell R, Palethorpe S Multitask Learning In Connectionist Speech Recognition Proceedings of the Tenth Australian International Conference on Speech Science & Technology: 8-10 December 2004; Sydney 2004 Canberra: Australian Speech Science and Technology Association Inc 312 315

[B14] Yuan X-T Yan S Visual classification with multi-task joint sparse representation Proceedings of the 2010 IEEE Conference on Computer Vision and Pattern Recognition (CVPR): 13-18 June 2010; San Francisco 2010 IEEE 3493 3500

[B15] Jacob L Vert J-P Efficient peptide–mhc-i binding prediction for alleles with few known binders Bioinformatics 2008 24 3 358 366 10.1093/bioinformatics/btm611 18083718

[B16] Widmer C Leiva J Altun Y Rätsch G Leveraging sequence classification by taxonomy-based multitask learning Research in Computational Molecular Biology 2010 Heidelberg: Springer Berlin 522 534

[B17] Yang W-H Dai D-Q Yan H Finding correlated biclusters from gene expression data Knowl Data Eng, IEEE T 2011 23 4 568 584

[B18] Puniyani K Kim S Xing EP Multi-population gwa mapping via multi-task regularized regression Bioinformatics 2010 26 12 i208 i216 10.1093/bioinformatics/btq191 20529908 PMC2881376

[B19] Calus MPL Meuwissen THE de Roos APW Veerkamp RF Accuracy of genomic selection using different methods to define haplotypes Genetics 2008 178 1 553 561 10.1534/genetics.107.080838 18202394 PMC2206101

[B20] Verbyla KL Hayes BJ Bowman PJ Goddard ME Accuracy of genomic selection using stochastic search variable selection in australian holstein friesian dairy cattle Genet Res 2009 91 5 307 311 10.1017/S0016672309990243 19922694

[B21] Habier D Fernando RL Kizilkaya K Garrick DJ Extension of the bayesian alphabet for genomic selection BMC Bioinformatics 2011 12 1 186 21605355 10.1186/1471-2105-12-186 PMC3144464

[B22] Zimin AV Delcher AL Florea L Kelley DR Schatz MC Puiu D Hanrahan F Pertea G Van Tassell CP Sonstegard TS A whole-genome assembly of the domestic cow, bos taurus Genome Biol 2009 10 4 R42 10.1186/gb-2009-10-4-r42 19393038 PMC2688933

[B23] Sargolzaei M Chesnais JP Schenkel FS Fimpute - an efficient imputation algorithm for dairy cattle populations J Dairy Sci 2011 94 E-Suppl. 1 421

[B24] Gilmour AR Gogel BJ Cullis BR Thompson R Asreml user guide release 3.0 Hemel Hempstead, HP1 1ES 2009 UK: VSN International Ltd

[B25] Brito FV Neto JB Sargolzaei M Cobuci JA Schenkel FS Accuracy of genomic selection in simulated populations mimicking the extent of linkage disequilibrium in beef cattle BMC Genet 2011 12 1 80 10.1186/1471-2156-12-80 21933416 PMC3224120

[B26] Grisart B Farnir F Karim L Cambisano N Kim JJ Kvasz A Mni M Simon P Frere JM Coppieters W Genetic and functional confirmation of the causality of the dgat1 k232a quantitative trait nucleotide in affecting milk yield and composition Proc Natl Acad Sci U S A 2004 101 8 2398 2403 10.1073/pnas.0308518100 14983021 PMC356962

[B27] Pimentel Eda C Erbe M Konig S Simianer H Genome partitioning of genetic variation for milk production and composition traits in holstein cattle Front Genet 2011 2 19 22303315 10.3389/fgene.2011.00019 PMC3268574

[B28] Su G Christensen OF Ostersen T Henryon M Lund MS Estimating additive and non-additive genetic variances and predicting genetic merits using genome-wide dense single nucleotide polymorphism markers PLoS One 2012 7 9 e45293 10.1371/journal.pone.0045293 23028912 PMC3441703

[B29] Toro MA Varona L A note on mate allocation for dominance handling in genomic selection Genet Sel Evol 2010 42 33 10.1186/1297-9686-42-33 20699012 PMC2928189

[B30] Vitezica ZG Varona L Legarra A On the additive and dominant variance and covariance of individuals within the genomic selection scope Genetics 2013 195 4 1223 1230 10.1534/genetics.113.155176 24121775 PMC3832268

[B31] Legarra A Misztal I Technical note: computing strategies in genome-wide selection J Dairy Sci 2008 91 1 360 366 10.3168/jds.2007-0403 18096959

[B32] Calus MP Right-hand-side updating for fast computing of genomic breeding values Genet Sel Evol 2014 46 1 24 24708180 10.1186/1297-9686-46-24 PMC4036606

[B33] Hayashi T Iwata H Em algorithm for bayesian estimation of genomic breeding values BMC Genet 2010 11 3 20092655 10.1186/1471-2156-11-3 PMC2845064

[B34] Meuwissen THE Solberg TR Shepherd R Woolliams JA A fast algorithm for bayesb type of prediction of genome-wide estimates of genetic value Genet Sel Evol 2009 41 1 10.1186/1297-9686-41-1 19284681 PMC2637029

[B35] Shepherd RK Meuwissen TH Woolliams JA Genomic selection and complex trait prediction using a fast em algorithm applied to genome-wide markers BMC Bioinformatics 2010 11 1 529 10.1186/1471-2105-11-529 20969788 PMC3098088

[B36] Li ZT Sillanpaa MJ Estimation of quantitative trait locus effects with epistasis by variational bayes algorithms Genetics 2012 190 1 231 249 22042575 10.1534/genetics.111.134866 PMC3249367

